# The Wildcat That Lives in Me: A Review on Free-Roaming Cats (*Felis catus*) in Brazil, Focusing on Research Priorities, Management, and Their Impacts on Cat Welfare

**DOI:** 10.3390/ani15020190

**Published:** 2025-01-12

**Authors:** Luana S. Gonçalves, Daiana de Souza Machado, Maria Eduarda Caçador, Giovanne Ambrosio Ferreira, Christopher R. Dickman, Maria Camila Ceballos, Fabio Prezoto, Aline Cristina Sant’Anna

**Affiliations:** 1 Graduate Program in Biodiversity and Nature Conservation, Center for Studies in Ethology and Animal Welfare, Federal University of Juiz de Fora, Juiz de Fora 36036-900, Brazil; dudacacador@hotmail.com; 2 Graduate Program in Experimental Psychology, Institute of Psychology, University of São Paulo, São Paulo 05508-030, Brazil; daianasm.dsm@gmail.com; 3 Aqualie Institute, Juiz de Fora 36036-330, Brazil; giovanneaferreirabio@gmail.com; 4 School of Life and Environmental Sciences, University of Sydney, Sydney, NSW 2006, Australia; chris.dickman@sydney.edu.au; 5 Faculty of Veterinary Medicine, University of Calgary, Calgary, AB T2N 4Z6, Canada; mariacamila.ceballos@ucalgary.ca; 6 Department of Zoology, Federal University of Juiz de Fora, Juiz de Fora 36036-900, Brazil; fabio.prezoto@ufjf.br; 7 Faculty of Agricultural and Veterinary Sciences, Department of Animal Science, São Paulo State University, Jaboticabal Campus, São Paulo 14884-900, Brazil; aline.santanna@unesp.br

**Keywords:** biodiversity, behavior, domestic cats, invasive species, animal welfare

## Abstract

Domestic cats (*Felis catus*) currently occupy the 38th place in the Global Invasive Species Database. Free-roaming cats potentially have broad-ranging impacts on wildlife, occupying most terrestrial environments globally as house pets, strays, or feral animals. In Australia, for example, cats are responsible for the decline in many vertebrate populations and extinction of several native mammals. However, in Brazil, few studies have explored either direct impacts of predation on wildlife, or other indirect impacts, such as competition for resources, niche overlap, hybridization, and disease transmission. In this review, we summarize and discuss 34 original research reports, published between 2001 and 2020, on impacts of free-roaming cats in Brazil. We briefly contextualize the history of cat domestication and present that, in Brazil, more studies have focused on the potential of free-roaming cats to transmit disease than to exert impacts via predation or competition on wildlife. More studies were conducted in Brazilian mainland areas (*n* = 23)—notably in Atlantic Forest—than on islands (*n* = 11). The review highlights potential impacts of cats on wildlife. We also discuss how control management strategies can affect the welfare of domestic cats, identifying potential knowledge gaps as well as opportunities for future research. Finally, understanding risks of cat predation is necessary to inform future measures to mitigate impacts on wildlife, without neglecting cat welfare.

## Simple Summary

The domestic cat (*Felis catus*) ranks 38th in the Global Invasive Species Database and has a widespread presence in terrestrial environments as pets, strays, or feral animals. Cats can also be companion animals with names and are often considered by people to be members of their family. Free-roaming cats significantly impact wildlife, causing species declines and extinctions, as reported in Australia. In Brazil, studies on their impacts are limited, focusing more on disease transmission than predation or competition. This review analyzes 34 research papers (2001–2020) on free-roaming cats in Brazil. There has been a greater focus on mainland areas, particularly in Atlantic Forest habitats. In addition to highlighting potential risks to wildlife, this review also considers the welfare implications of cat management strategies. Understanding cat predation and areas of occurrence is vital for developing effective conservation measures that balance biodiversity protection and animal welfare.

## 1. Introduction

Invasive alien species are animals, plants, or other organisms introduced by humans, intentionally or accidentally, into places outside their natural range, negatively affecting native biodiversity, ecosystem services, or human economy and well-being [1]. According to the International Union for Conservation of Nature, 1 in 10 species on the Red List of Threatened Species is imperiled by invasive alien species [1]. One of the most widely distributed of these invasive aliens is the domestic cat (*Felis catus* Linnaeus, 1758) [2], occupying 38th place on a globally invasive species list [3]. Paradoxically, the domestic cat is not just an invasive alien species but also a favored companion animal for people in all parts of the world to which the cat has been introduced [4]. Pet domestic cats have their food, shelter, veterinary care, and other needs intentionally met by their guardians [5]. Stray cats obtain resources by exploiting anthropogenic resources in cities [6,7,8]. A feral cat is a mostly self-sustaining cat capable of surviving with or without direct human intervention [9,10,11]. A more general term, “free-roaming” (or “free-ranging”), is sometimes used to describe domestic cats that have access to outdoor environments and potential to affect native species [5]. Such cats may be stray or feral or even unconstrained house pets. Here, we review the impacts and management of cats in Brazil and, because of its generality, we use the term “free-roaming” to encompass all relevant cat-related studies.

Free-roaming female cats achieve sexual maturity within 1 year of age, cycle continuously until successfully mated, and give birth to 2–3 litters of 1–7 young per year, following 55–65 gestation days [12,13,14]. This fecundity ensures high potential recruitment into local areas and increases the likelihood of encounters between cats and native species. Many threatened native species are now restricted to urban and suburban environments or to patches of natural vegetation [15], while many further endemic species are restricted to small islands [16]. Free-roaming cats have potentially deleterious impacts on native species in all these situations.

In the first instance, cat impacts may arise via direct predation. Cats are opportunistic carnivores that prey on various animal species, depending on their availability in the environment [17,18,19]. Although small terrestrial mammals and birds often predominate in the diet [20,21], reptiles, amphibians, and invertebrates [22] can be preyed upon by cats. These differences in diet depend more on the ecosystem where the study was conducted and the methodology used than on the pattern of depredated species [23]. Some cats may continue to prey on wildlife even when food is provided by humans [24].

In many island environments, such as on oceanic islands, introduced cats occupy most available habitats due to the lack of native predators, and may have dramatic impacts on prey populations because island species do not have defense mechanisms against novel predators [25]. Island populations of several native mammal species have been depleted by cat predation [26]. In a review conducted in 2011, the authors reported that feral cats on islands worldwide have impacted 175 vertebrate species, the majority of which were endemic [27]. They also stated that cats were responsible for at least 14% of global extinctions of birds, mammals, and reptiles, and represented the primary threat to nearly 8% of critically endangered birds, mammals, and reptiles [27]. Further extinctions of native vertebrates have been documented in mainland environments where cats have had an active role in driving species to extinction. However, other vertebrates, such as the European red fox, also contributed to extinction [28]. Given that most studies on this topic have been conducted in Australia, it is well documented that 30 mammal species have gone extinct since 1788, with predation by feral cats and red foxes identified as the primary factors contributing to the extinction of these species or their decline [29]. At a global scale, a review published in 2015 reported that 25.22% of 2084 species preyed upon by free-roaming cats are of conservation concern on islands, and 8.62% of species are at risk in continental areas, with South America being one of the continents with fewest studies on the topic (25), in contrast to Oceania, with many studies in Australia (215) [28]. Recently, Lepczyk et al., 2023 [23], conducted a literature review, including both oceanic islands and continents, and produced a comprehensive quali-quantitative synthesis of relevant scientific publications on impacts of cats on wildlife. The geographical concentration of studies on developed-world nations continues, with most studies on continental areas conducted in Australia (37.1% of 186 studies), Europe (28.5%), and North America (25.3%). However, information from the highly biodiverse continents of Africa (3.2% of studies), Asia (1.6%), and South America (4.3%) remains scarce, revealing a concerning knowledge gap. Another noteworthy finding was the species bias, as most studies (56.9%) focused on impacts of cats on specific taxonomic groups rather than considering all potentially affected wildlife. Most studies focused on birds (47.1%) and mammals (36%), whereas amphibians (1.1%), invertebrates (0.5%), reptiles (4.2%), and fish (0%) received minimal attention. Studies addressing more than one taxonomic group accounted for 11.1%.

In addition to direct impacts of predation on wildlife, cats can generate indirect impacts, e.g., via competition and spatial overlap [30], hybridization [31], or transmission of diseases [32,33]. Direct contact among domestic animals, people, and wildlife is linked to zoonotic spillover risk, although the potential threat posed by zoonoses is often poorly recognized by human populations, as reported in a questionnaire study conducted in Brazil [34]. For example, cats introduced on islands, such as Fernando de Noronha, Pernambuco State, Brazil, may harbor lineages of the protozoan parasite *Toxoplasma gondii* that differ from those on the mainland [35], posing immunological risks to cats and people. Cats can act as reservoirs for arthropod-borne pathogens [36] and harbor a variety of gastrointestinal parasites, bacteria, and protozoan pathogens, including *Ancylostoma* sp., *Strongyloides* sp., *Trichuris campanula*, and *Toxocara cati*. This highlights the potential risk of parasitic infections to the local human population, exacerbated by the presence of both feral cats and rodents in the archipelago [37].

Most research on impacts of cats on wildlife has been conducted in Australia [38,39,40,41], the United States of America [42,43], and Europe [44,45]. Scientific information about cat impacts in South America is scarce [23], especially in the largest country of the continent, Brazil. Currently, Brazil has 489 known invasive alien species; the cat presence is reported consistently in many areas, albeit mostly incidental sightings [46,47]. Thus, there are many gaps regarding impacts of domestic cats on wildlife, especially in areas close to green fragments, such as conservation units, parks, and/or reserves in Brazil. There is, in addition, notably, limited research on cats as an invasive alien species in Brazilian island environments, where risks to native species and biodiversity are likely greatest [48,49,50].

In Brazil, there are 524 mammal species (of which 131 are endemic), 517 amphibians (294 endemic), 1622 birds (191 endemic), and 468 reptiles (172 endemic), as well as approximately 3000 species of freshwater fish and an estimated 10 and 15 million species of insects [51]. Brazil is also home to > 27 million pet cats, with a significantly larger number of stray or feral cats likely present [52]. Since cats prey on a wide range of animals, including small mammals, birds, and invertebrates [53,54], this rich wildlife biodiversity is at significant potential risk from cat-related impacts. To our knowledge, in Brazil, there is only one recent review addressing impacts of domestic cat predation on vertebrates [55]. However, this review did not address potential indirect impacts of cats (e.g., disease transmission) and cat’s welfare, and moreover, control and/or management measures were not detailed. Therefore, in the present review, our objective was to gather the relevant literature on direct and indirect impacts of free-roaming cats on wildlife in Brazil. Furthermore, we use the information gathered to identify priorities for future studies and management actions, associating these strategies with animal welfare principles. Our intention is not to portray cats as a villain for wildlife conservation but rather to position them as a focal species for further studies, in both Brazilian and global contexts.

## 2. The Brazilian Context

Brazil is the largest country in Latin and South America and the fifth largest country in the world (in area and population). The national territory extends almost 4400 km (2732 miles) from north to south (5°16′10″ N to 33°45′03″ S latitude), and 4320 km (2685 miles) from east to west (34°47′35″ W to 73°58′59″ W longitude). According to data from the Brazilian Institute of Geography and Statistics [56], there are 125,251 animal species known in Brazil. This richness, however, results in potentially serious conservation concerns. Brazil is the largest country in terms of the number of endangered fauna species (1249), according to the recent update of the Brazilian Ministry of Environment and Climate Change [57]. One of the main factors associated with the loss of species is widespread habitat loss due to clearing of native vegetation for human land use, a process that leads to further deleterious environmental changes, such as climate change [58,59,60]. Furthermore, climate change facilitates spread and establishment of many alien species and creates opportunities for them to become invasive [61].

The few studies exploring impacts of cats on wildlife have been conducted mostly in the Atlantic Rainforest biome, the most threatened biome in Brazil [21,24,36]. Fewer studies have been conducted on islands. Much of what is currently available in the literature on this subject in Brazil was carried out on Ilha Comprida, São Paulo State, where impacts of cat predation on wildlife have been analyzed [24,50,54]. Further studies on the islands of Fernando de Noronha have focused on impacts related to both feral and pet cats on domestic animals and wildlife arising from infection by zoonotic diseases, especially the protozoan parasite *T. gondii* [62,63].

Concern about cats (and dogs) as invasive species is not new in Brazil [48,49]. However, studies on these carnivores are progressing slowly, perhaps because there is a lack of information about them as drivers of biodiversity loss. Conversely, in countries such as Australia, this topic has been well researched [64,65,66], leading to adoption of prevention and control strategies [67,68,69,70,71], recognizing the need to mitigate threats posed by both pet and feral domestic cats. Brazil should not delay recognizing potential threats that cats pose to wildlife and should adopt a precautionary approach in dealing with them [72]. Furthermore, strategies to reduce the risks of cats to wildlife must consider cats’ welfare, as this is a major concern of humane societies. Our review provides a step in this direction.

## 3. Literature Search

To compile articles for this review, we selected research studies conducted in Brazil using the following keywords: “predation” AND “cat” AND “Brazil” AND “zoonoses”. We used electronic databases, Scopus, Web of Science, and Google Scholar, to locate articles. Only studies published in English that were conducted in Brazil and had the domestic cat as a focal species were selected, excluding review articles, monographs, master’s dissertations, doctoral theses, and book chapters. Articles were assessed based on the following: (1) city and/or state where the research was conducted, (2) whether on Brazilian mainland or islands, (3) biome, (4) main prey, (5) methodology (including field, laboratory, and analytical methods), and (6) nature of the cat impact(s) that was studied (Supplementary Material Table S1).

**Figure 1:**
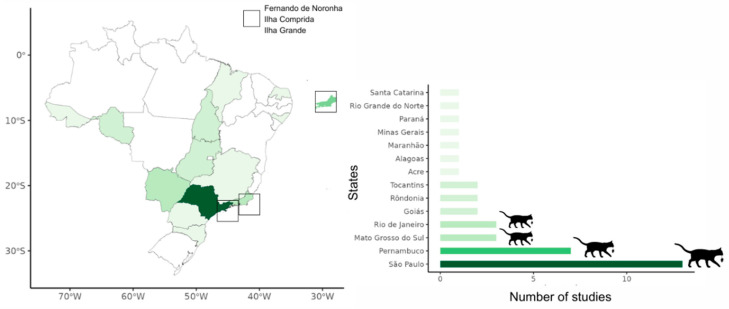
Number of studies addressing cat impacts on wildlife in Brazil (by state). The larger the cat–prey icon, the greater the number of studies on this topic in that region.

In total, 34 research articles were retrieved, with 11 conducted in Brazilian island environments and 23 focused on continental areas (Figure 1). Two of the 34 studies [73,74] were not included in the state-based map (Figure 1), as they were conducted nationally.

**Figure 2:**
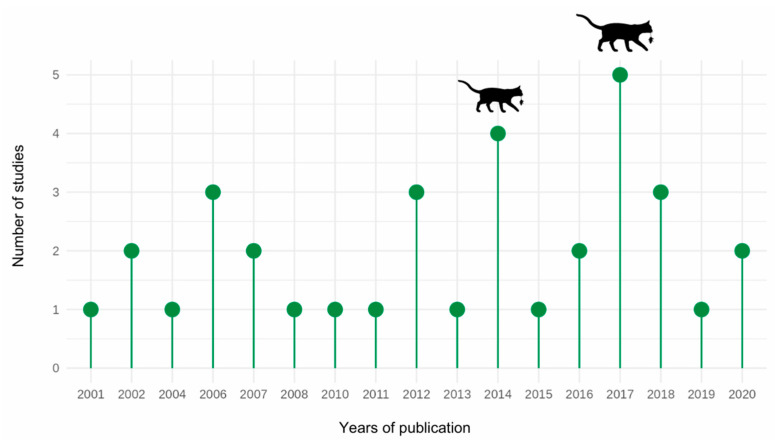
Number of studies addressing cat impacts on wildlife in Brazil (by year of publication). The larger the cat–prey icon, the greater the number of studies on this topic in that year.

Most (73.52%, 25/34) studies focused on cat disease transmission using serological or molecular methods, with fewer studies assessing cat impacts via predation—primarily on small terrestrial mammals—and spatial overlap or resource competition with another biota. These articles were published between 2001 and 2020 (Figure 2). In the present literature review, we discuss potential impacts—both direct (predatory) and indirect (disease transmission, spatial overlap)—of free-roaming cats on wildlife and domestic fauna in both insular and mainland environments of continental Brazil. The impacts are discussed in the context of global research before exploring management options and their impacts on cats’ welfare in Brazil.

## 4. Risks and Conflicts Between Cats and Wildlife in Brazil

### 4.1. Predation in Island Environments

Studies on potential impacts of domestic cats in Brazilian island environments have been conducted on Ilha Comprida (four studies), Fernando de Noronha (six), and Ilha Grande (one). As an opportunistic carnivore, the domestic cat adapts its hunting behaviors to its surroundings. On Ilha Comprida, a fishing island in São Paulo, researchers analyzed feces and prey remains brought home by cats (*n* = 482). Despite being fed by their owners, these cats exhibited a diverse diet and opportunistic predation habits. Their primary prey included invertebrates (54.97%), mammals (34.5%), and birds (9.94%, predominantly from the Passeriformes order), with amphibians comprising a minor proportion (0.58%) [24,54]. Given that Ilha Comprida serves as both a nesting site and a migratory route for various bird species, evidence of predation on migratory birds was expected. However, as not all bird species were identified in their study, the authors did not rule out the likelihood of predation on migratory birds outside the Passeriformes group [54].

On other more sparsely inhabited islands, such as Fernando de Noronha, the potential impact of cats on migratory birds was reported as being due to both predation and changes in migration routes that cats appeared to induce. Many cats (*n* = 1287) were reported on the island [49], and they have been recorded chasing and preying on seabirds [21]. The presence of these predators in locations called “stopovers”—places chosen by migratory birds to build their nests or replenish depleted food resources—can interfere with migratory activity, leading birds to choose other routes to traverse. In addition to birds, cats have been recorded preying on turtle nests, rodents such as Moco (also known as rock cavy, *Kerodon rupestris*), and reptiles, e.g., lizards of the skink genus *Mabuya* [21]. Close co-existence between cats and residents of Fernando de Noronha has prompted several studies on potential health risks on the island. Research has mainly concentrated on identifying protozoan parasites like *T. gondii* [74] and *Neospora caninum* [75]. These studies aimed to understand dangers that the feline population may pose to humans and wildlife, highlighting broader ecological impacts of cats in island environments. This topic is discussed further below (Section 4.2).

The population of domestic cats in Ilha Grande, Rio de Janeiro State, was investigated between May and November 2008, including an estimation of predation and efficiency of population control methods [48]. The researchers projected cat population growth over 50 years and reported that the population could be reduced only if at least 70% of females were spayed or removed annually. They determined that the island had a high density of cats (616 cats/km^2^). In addition, most cats recorded on the streets did not have an owner. The study identified that owned, but still free-roaming, cats brought home 93 prey items (average)—invertebrates (54), mammals (18), birds (11), reptiles (6), and amphibians (4)—representing a high dietary diversity [48].

### 4.2. Transmission of Diseases in Island Environments

Interactions between domestic cats and wildlife are not limited to predation; encounters can also lead to pathogen-facilitated transmission and circulation, potentially resulting in spillover. The main pathogen studied on Brazilian islands is the protozoan *T. gondii* [76,77]. This is an obligate intracellular protozoan whose definitive host is the domestic cat, responsible for transmitting the pathogen through the release of oocysts in its feces that are picked up subsequently by secondary hosts [78]. This relationship is well established, as the absence of *T. gondii* has been documented in regions with no cats, highlighting the importance of felines in transmission of the disease [78].

In Brazil, the occurrence of *T. gondii* in the jaguar *Panthera onca* has been described [79]. In a study conducted in 2015, antibodies were detected in all jaguars sampled in three major habitats (Cerrado, Pantanal, and Amazon Forest) [79]. Similar results were reported on islands. On Fernando de Noronha Island, *T. gondii* antibodies were detected in 170 of 231 (73.6%) sampled wild animals (including the first record in the order Pelicaniformes, the western cattle egrets *Bubulcus ibis*) and 49.5% of sampled domestic animals [62]. A few years later, additional studies were conducted in the same location, with an increase in the prevalence of *T. gondii* infection in cats, dogs, pigs, and horses, with antibodies identified in 71.26% of domestic cats sampled [63]. In this study, the authors highlighted the transmission of the disease from felines to humans and farm animals, indicating a higher prevalence of infection in dogs, pigs, and horses in areas with a high prevalence of seropositive cats. Most of the studies on this topic in Brazil were concentrated on Fernando de Noronha Island, the largest Brazilian oceanic island, with important biological diversity [35,37,62,63].

Another well-studied cat disease is caused by the feline immunodeficiency virus (FIV), already identified in domestic cats and wild cats [80]. Brazilian wild felines have also been reported to carry antibodies of feline herpesvirus and parvovirus, detected in oncilla *Leopardus tigrinus*, ocelot *L. pardalis*, and cougar *Puma concolor* [81], with rabies and distemper in *Panthera onca* [82]. The proximity of domestic cats with wild felines can spread diseases from the wild to the domestic environment, or vice versa [83,84]. This is even more concerning in adult male non-neutered cats with outdoor access. Due to their large home range, driven by mate search and elevated testosterone, the likelihood of territorial and mating-related conflicts increases, raising risks of disease transmission [85,86,87,88].

Antibodies against feline leukemia virus (FeLV) were detected in two free-living *Puma concolor* individuals within the Pantanal and Atlantic Forest biomes, although the viral antigen was not identified [81]. Similarly, the presence of the FeLV antigen in two domestic cats near Cantão State Park was reported, but there was no evidence of the virus in jaguar populations in the studied areas [82].

In a broader context, Levy et al. (2006) [89] reported that 2.3% of sampled cats in the USA and Canada were seropositive for FeLV, 2.5% for feline immunodeficiency virus (FIV), and 0.3% for both. Multivariate analyses highlighted significant associations between seropositivity and factors such as age, sex, health status, lifestyle, and origin of the cats. Adult cats were more likely to be seropositive than younger cats, with sexually intact adult males having higher seropositivity than females. In addition, outdoor cats that were ill at testing were more frequently seropositive than healthy indoor cats [89].

Cats can also act as vectors of zoonotic diseases [90,91]. Pathogen transmission can represent a serious threat to wild felid species [81,82,89], being a main cause of wild population decline and, therefore, representing an important threat to biodiversity.

### 4.3. Spatial Overlap and Hybridization in Island Environments

Wildlife behavioral changes have been documented in environments frequented by domestic cats, influenced by scent marks in cat urine and feces [92]. Especially in inland environments, introduction of a new predator alters selective pressures on prey populations, potentially leading to changes in their behavioral repertoires [92].

One of the most important concerns related to encounters between opportunistic mesopredators and other animals is spatial overlap and, in some cases, competition [93]. Spatial overlap occurs when two species coexist in the same habitat, and competition can occur if they use the same resources [94]. Introduced animals tend to compete with wild animals mainly for food and habitat, increasing this influence when resources are limited [95]. In a study on Ilha Comprida, São Paulo State, Brazil, overlapping areas used by domestic cats and small, medium, and large wild felines were observed, suggesting potential intraguild competition [30]. The authors indicated that intraguild competition between these species groups may have an important role in structuring habitat use, since, typically, wild cats tend to have lower adaptability compared to domestic cats, being generally fewer in number and, therefore, at a disadvantage in frequency-dependent encounters [30]. Competition does not only occur within the same animal group. Other groups can also be affected by introduction of a new species, such as carnivorous mammals, birds, and reptiles [96].

Birds’ migratory routes can also be severely impacted by the presence of introduced species. Typically, birds select sites to stopover that allow them to forage efficiently and replenish energy stores, but in locations with domestic animals, this behavior may be altered [97,98]. In Barra Grande Mangrove Environmental Protection Area, Ceara, Brazil, the greatest impacts on migratory birds are caused by domestic cats or dogs scaring away resting or feeding adults [99,100]. In addition to direct impacts, domestic animals can interfere by reducing the tolerance of birds to human disturbances, causing a range of behavioral changes, such as anti-predatory behavior [101].

Hybridization is another relevant process that is likely to occur between wild felines and domestic cats [102]. Spatial overlap can compromise the integrity of wild cat populations [102,103], directly threatening their genetic integrity [104]. One of the best-studied examples is the hybridization that occurs between domestic cats and Scottish wildcats *Felis sylvestris* [104], with introgression of domestic traits into wild cats, considered a key threat to their conservation [105]. In Brazil, however, there is no scientific evidence of hybridization between domestic and wild species [106].

### 4.4. Predation and Spatial Overlap in Continental Environments

Although more studies have occurred on the continent than on islands, most have been concentrated in southeastern Brazil, mainly in São Paulo State, in natural, urban, and rural environments [107,108]. Rural and urban environments provide a variety of prey species for cats. For example, bats are common in urban and rural areas, as demonstrated by Costa-Pinto et al. [109], who reported predation on the pale spear-nosed bat *Phyllostomus discolor* by cats in an urban environment in São Paulo city, São Paulo State. This is problematic for species conservation, but mainly in relation to public health. Some hematophagous bat species host the rabies virus, a deadly zoonosis that can be transmitted to humans by cat bites and/or scratches. A one-decade study of patient-bite data from the National Health Information System in Brazil determined that the incidence of bites from cats and bats had increased over time, whereas bites from dogs and herbivores remained relatively stable [73]. In addition, the most frequently reported bites from domestic and wild animals occurred in urban environments (71%) [73].

Aside from the observations noted above, only one other study of cat predation has been conducted in continental Brazil, reporting that there are more free-ranging cats in urban environments than free-ranging dogs, whereas in rural environments, the numbers of free-ranging dogs and cats are similar [53]. The study was carried out around the University of São Paulo (USP), Piracicaba campus, Piracicaba, São Paulo State, and used scat analysis to evaluate dog and cat diets. Cats preyed largely on invertebrates (63.2%) and mammals (57.1%), with an estimated mammal consumption between 2.01 and 2.9 kg/year/cat. In Brazil, particularly in peri-urban and rural areas like the study site, there is no widespread enforcement of laws requiring owners to retrieve dog feces, and many dogs roam freely, even if they have a home. This facilitates scat collection and analysis. This situation also applies to free-ranging cats. These analyses, as well as places where predation events occur, are important for establishing management programs aiming to mitigate predation on wildlife by domestic animals such as cats [53].

Another aspect to consider is related to indirect, or compensatory, predation. A major cause of mortality for some bird species arises from accidents, particularly collisions with glass windows. Although few studies have explored this, some authors suggest that cat predation may be compensatory rather than additive. Birds depredated by cats are often already vulnerable, either due to a previous trauma or illness. Therefore, predation could be compensatory and not significantly increase the total number of deaths that would likely have occurred. For instance, Baker et al. [110] reported that approximately 60% of monitored cats over a year at five sites in the city of Bristol, UK, never brought prey back home. Despite this, the estimated number of birds killed was substantial. This aligns with the assertion that cat predation represents compensatory mortality, as it primarily targets weakened individuals, rather than increasing overall mortality [110].

### 4.5. Transmission of Diseases in Continental Environments

The protozoan parasite *T. gondii* has been widely studied in Brazil, probably due to its high prevalence in domestic and wild animals and humans [111,112,113]. Diagnostic techniques have advanced over the last two decades, with increased specificity and sensitivity to *T. gondii*. However, increases in deforestation and urbanization, and, consequently, proximity of domestic animals to wildlife have also generated a more favorable environment for maintenance, release, and dissemination of infectious diseases [78].

Although clinical toxoplasmosis is uncommon in felids, infection with *T. gondii* is frequent. Young or old animals with compromised immune systems may develop the disease [114,115,116]. Despite the rarity of the clinical form, infected felids disseminate the parasite into the environment, exposing other animals, especially Neotropical primates that are highly susceptible to toxoplasmosis and may succumb to it [117,118,119]. Thus, control of toxoplasmosis in felids is crucial for biodiversity conservation [120].

Most studies on toxoplasmosis in Brazil used serological techniques, e.g., indirect immunofluorescence antibody test (IFAT) that aims to identify seropositive animals based on antibody titers [121,122,123]. Other studies assessed the prevalence of *T. gondii* using a modified agglutination test (MAT) [79,124] or molecular analysis to detect *T. gondii* DNA, through molecular amplification (PCR) [120].

One study investigated the genetic diversity of *T. gondii* in Brazilian wild felines using the PCR-RFLP (Polymerase Chain Reaction–Restriction Fragment Length Polymorphism) technique. This technique combines specific DNA amplification with digestion by restriction enzymes, enabling identification of variations in the parasite’s genetic sequence. Through PCR-RFLP, researchers identified a high diversity of *T. gondii* genotypes, including atypical variants, highlighting the complex epidemiology of toxoplasmosis in continental environments [124]. The study also confirmed that wild felines such as *Leopardus geoffroyi* and *Leopardus tigrinus* are hosts of *T. gondii*. Due to the overlap of their habitats with residential areas, these felines can contribute to transmission of the disease to domestic animals and humans. Understanding interactions between wild and domestic felines, coupled with detailed characterization of *T. gondii* genotypes using PCR-RFLP, is essential for development of more effective control measures for toxoplasmosis [124].

In addition to toxoplasmosis, some studies in Brazil have reported a high prevalence of *N. caninum* and *Leishmania* spp. in cats [125,126,127,128]. Most studies performed serological tests for *T. gondii* and *N. caninum*, as lesions are similar [110,122]. Cats with outdoor access or urban vegetation can prey on various animals, including pigeons [129,130] and rats [131]. These prey animals are part of the parasite life cycle; therefore, cats can become infected with both types of parasites through predation.

Leishmaniasis (*Leishmania * spp.) is a zoonosis that has been extensively studied in dogs, as they were originally considered the only domestic host [132]. However, cats appear to have an important epidemiological role in the leishmaniasis cycle. The first case of American visceral leishmaniasis in cats was reported in 2008 in Rio de Janeiro State, with a seroprevalence of 25% [132]. After 2008, cat seroprevalence percentages have been reported in other places, including 0.7% in the city of Araçatuba, São Paulo State [125], and 4.2% in the city of Andradina, São Paulo [107]. More studies in Brazil are urgently needed, especially in *Leishmania*-endemic areas as, by harboring the virus, cats are a potential disease risk for humans.

Zoos are mostly located in urban environments and often accessed by stray cats. Cats can act as reservoirs for ectoparasites, such as fleas and ticks, which, in turn, are carriers of pathogens. Haemoplasmas and *Bartonella* are the most common bacterial diseases transmitted by these vectors [133,134]. In a study of the prevalence of these diseases in blood samples of 37 free-roaming cats in a Brazilian zoo [36], there were 12 (32%) positive cats for *Haemoplasma* and 11 (30%) positive cats for *Bartonella*. In that study, molecular analyses of various pathogens were conducted on stray cats that lived at the *Fundação Parque Zoológico de São Paulo*, São Paulo, in southwestern Brazil [36]. In the northeastern region of Brazil, the prevalence was reported as 15% using the IFAT test and 53.3% using PCR on *Ctenocephalides felis* (fleas) collected from cats [135].

In rural environments, domestic animals are usually closer to wildlife; this interaction can promote pathogen transmission [136]. A high prevalence of endoparasites in fecal samples (*Eimeria* spp., *Giardia* cysts, *Ancylostoma*, *Toxocara*, *Sarcocystis*, *Ascaridida*, and *Capillaria* spp.) was reported in domestic animals (197 cattle, 37 horses, 11 sheep, 25 swine, 21 dogs, one cat, and 62 groups of chickens) evaluated in rural properties in the municipality of Teodoro Sampaio, São Paulo State. However, only eggs of roundworm *Ancylostoma* spp. were reported in the cat sampled in this study, which could be attributed to the low sample size (one cat). Although there are more studies regarding cat pathogens in continental Brazil than on other topic areas, there is still much to study, especially as pathogen prevalence is likely to change with changing environmental conditions, and recent improvements in assay methods provide more accurate results than in some earlier studies [136].

## 5. Free-Roaming Domestic Cats Need to Be Managed, but the Question Remains: How?

The number of free-roaming domestic cats is growing in many parts of the world, as unconstrained pets, as strays, and as members of feral populations, with potentially deleterious consequences for wildlife and other biodiversity components. In Australia, cats are considered one of the main threats to wildlife, particularly when viewed as an invasive alien species [38,39,40,41]. Conventional strategies such as home containment, sterilization of strays, and feral cat culling are frequently implemented, but often with limited effect. Controlling domestic cat populations in regions where they are a threat is challenging for the scientific community and the broader population. Cats are highly valued companion animals, often considered part of the human family by their guardians [34]. Furthermore, many people are unaware of, or do not care about, the problems free-roaming cats represent to wildlife and continue allowing their animals to go beyond the walls of their homes or to foster colonies of stray cats. These realities impose difficulties in identifying how impacts of domestic cats can be effectively reduced or managed.

### 5.1. Non-Lethal Strategies

#### 5.1.1. Responsible Ownership

Responsible ownership of pet cats is the principal strategy to mitigate their impacts on wildlife. However, it is not simple to ensure responsible ownership. Some people, guardians or not, believe that cats need to live without restrictions on their home range, and that unconstrained movements improve cats’ welfare. To reinforce their point, they argue that cats are a species that need to hunt or that their house does not provide physical or structural conditions to limit cat movements [137,138]. Some cat guardians also suggest that outdoor access offers cats greater opportunities for physical and mental stimulation as this provides a less monotonous and less predictable environment, allowing them to exhibit natural behaviors such as hunting, exploring, and climbing [138].

The risks and benefits associated with indoor and outdoor management for cats have already been studied in various parts of the world [139,140,141]. Rochlitz inferred that welfare risks associated with restricted environments in cats include inactivity and obesity, household hazards (e.g., accidents, poisoning), and behavioral problems (e.g., inappropriate elimination/toileting), among other situations without a potential risk of death. However, risks associated with outdoor access include infectious diseases (viral, bacterial, parasitic, etc.), road traffic accidents, dog(s) and other animal attacks, and poisoning; all situations with an imminent risk of death [142]. Other studies have reinforced the points raised by Rochlitz [143,144]. A study on the welfare of stray cats over 3 years in Japan reported that approximately 80% of cats had disappeared by the end of the study. The authors suggested that the most likely cause for the disappearance was death from infectious diseases and injuries from fights [145].

In Brazil, one study reported that outdoor access was associated with a higher risk of contracting sporotrichosis (a skin infection caused by the fungus *Sporothrix*), disappearances, mistreatment, car accidents, and poisoning [34]. Studies in São Paulo city, São Paulo, reported that cats are more often victims of carbamate poisoning than dogs [143,144]. This primarily arises because many people do not tolerate cats visiting their homes or gardens, leading them to the cruel practice of setting poison baits. Additionally, there are occurrences of highly cruel practices with cats and dogs, like drowning and burning [143,144]. For all these issues, indoor management should be the best alternative. This prevents several risks to the welfare of both cats and wild animals and also prevents unwanted offspring and, consequently, overpopulation of free-roaming or feral cats [146,147].

There are other strategies that cat guardians could use in addition to restricting cats’ outdoor movements, including neutering and the use of accessories that can prevent predation by cats, such as Birdsbesafe^®^ and Catbibi^®^ [148,149]. One study tested various non-invasive strategies to prevent hunting, including: (i) fitting cats with Birdsbesafe^®^, which are brightly colored collars; (ii) providing feed with high meat-protein content; (iii) providing dry food that was available via a puzzle feeder; and (iv) guardians using toys and interacting with cats for a minimum of 5 minutes a day [150]. These strategies reduced the number of prey animals captured and brought home by cats in households where foods rich in meat proteins were provided (36%), and in households where 5–10 min of daily owner–cat play was introduced (25%). The use of Birdsbesafe^®^ bibs reduced the number of birds captured and brought home by 42% but did not reduce this for small mammalian prey [150]. Thus, non-invasive measures that are likely supported among cat guardians can be performed to reduce hunting behavior, as they do not have evident negative implications for cat welfare [150]. However, since hunting is often perceived by many guardians as a natural behavior for cats, some are not motivated to prevent or minimize it. In some cases, cats are even kept specifically for their ability to hunt species perceived as pests, e.g., rats and other rodents [151,152].

In Brazil, there is a significant direct correlation between cat guardian awareness and educational level and the guardian keeping their cat(s) indoors [34,137]. Guardians with higher levels of education have more available information and tend to have a closer relationship with their animals, with a greater frequency of responsible ownership practices [34,137]. Therefore, it is essential to provide guardians with high-quality information to promote responsible practices for care and management of their domestic cats. This information should include not only impacts of cat predation on wildlife but also risks of allowing a cat to roam freely. By highlighting these risks, even guardians unconcerned with predation may be encouraged to keep their cats indoors. Additionally, it is essential to include viable alternatives and solutions, such as collars and devices that reduce predation, sterilization to control the feline population, and microchips for identification. Guardians’ education and awareness are essential to understanding and taking responsibility for protecting wildlife and the welfare of their cats. Based on research in other places, people who are informed about potential impacts of their cats on wildlife, and risks associated with outdoor management, are more likely to be responsible guardians [153].

#### 5.1.2. Trap–Neuter–Return (TNR)

Trap–neuter–return (TNR) is considered an alternative to lethal control methods for free-roaming cats in some jurisdictions. This strategy originated in Europe in the 1950s and has spread globally. Free-roaming cats are captured, neutered, vaccinated, dewormed, and returned to the capture site, often alongside efforts to promote their adoption. TNR is primarily implemented for stray cats without ownership and aims to reduce the number of free-roaming cats and their associated impacts, including harm to wildlife, community nuisances, and disease transmission [154].

TNR is highly controversial in the scientific literature. Some authors argue that it lacks effectiveness and is resource intensive, with cat adoption rather than *TNR* being the critical factor in reducing populations [155]. Conversely, others believe TNR is effective but emphasize the need for long-term studies to quantify its success [156,157,158].

Several questions remain, mainly related to cat welfare and risks of TNR to wildlife and people [159], and remain a challenge for future studies. The use of TNR in stray and feral cat colonies on two university campuses (Salto and Itu) in São Paulo State, Brazil, reported an annual sterilization rate of 75 and 68%, respectively, over 2 years. Cats were neutered, dewormed and given an anti-rabies vaccine, and kittens were put up for adoption [160]. Despite this initiative, the number of cats involved in the program was low compared to the overall stray cat populations, and the programs would need to be implemented for longer intervals to provide reliable assessments of costs and benefits.

Because of conflicting and often poorly supported results, the efficiency of TNR remains controversial, and there is a need for more studies evaluating its long-term effects. Additionally, TNR does not address the lethal impacts of cats on wildlife; inevitably, many cats remain free to roam in any TNR program and continue to be exposed to wide-ranging risks, as mentioned above.

### 5.2. Lethal Strategies

**Figure 3:**
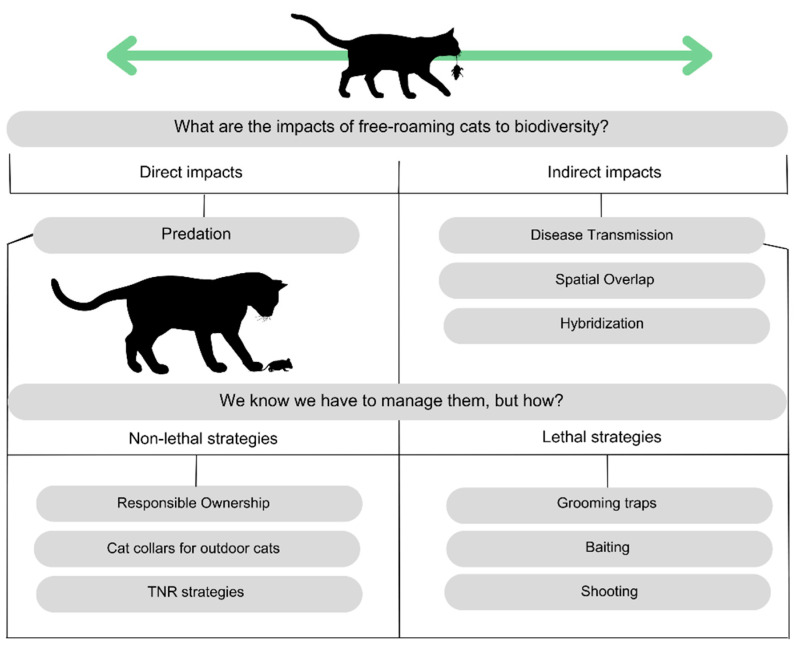
Biodiversity impacts and management strategies of free-roaming cats, where: TNR = trap–neuter–return.

In situations where cats have highly negative effects on wildlife or other assets, interventions at the population level have been used in some parts of the world, such as eradication with lethal tools—shooting, baiting, or grooming traps. Such interventions have been used on feral cats to reduce their impacts on threatened and endemic vertebrate species in Australia [161,162]. These management strategies are summarized in Figure 3.

Another relevant point concerns removal of invasive mammals, which paradoxically can sometimes endanger native prey species. For example, there is a dynamic interaction among prey (birds), mesopredators (rats), and superpredators (cats), first discussed by Courchamp et al. [163] and later updated in a model by Fan et al. [164] as the “bird–rat–cat” model. In this context, two scenarios are described: (1) severe mesopredator release, where suppression of superpredators leads to an explosion of mesopredators, resulting in extinction of their shared prey; and (2) mild mesopredator release, where mesopredators negatively impact endemic prey populations but do not drive them to extinction.

For example, on a marine island, removal of cats and rats facilitated the persistence of a native predator, the Atlantic Central American milk snake *Lampropeltis polyzona*. Normally preyed upon by rats, these snakes, in the absence of their predators, were able to consume large numbers of bird eggs. These snakes depredated~ 40% of the hatchlings in a booby colony annually within a single hectare [165]. In summary, eradication of introduced superpredators like feral domestic cats, as modeled in the “bird–rat–cat” framework, is not always the best solution for protecting endemic insular prey species. Careful consideration of complex ecological interactions is essential to avoid unintended consequences.

In Brazil, there is no law authorizing euthanasia of healthy animals for control purposes. Euthanasia is permitted only in cases of serious, incurable disease and/or situations that pose a risk to other animals and public health and should be performed by a registered veterinarian (Law 14,228/2021) [166]. The Law also highlights the prohibition of the elimination of dogs and cats by zoonosis control bodies, public kennels, and similar official establishments. However, a specific exception may occur on the island of Fernando de Noronha where feral and stray cats depredate various native or endangered animal species such as *Mabuya* spp. and various landbirds (see Section 4.1) [167]. Currently, there is an Action Plan for controlling cats on Fernando de Noronha; this Action Plan provides information for the general population about how they could control their cats. The plan also proposes conducting a public consultation about the non-application of State Law 14,139/2010 [168] in Fernando de Noronha that currently prohibits euthanasia of healthy cats. However, this Action Plan was published before the effective date of Federal Law 14,228/2021 [168], and, therefore, this public consultation document may no longer be accepted. A district decree was recently published to limit the entry and stay of domestic animals on Fernando de Noronha [169]. This decree prohibits the entry of cats and other domestic animals, except in some very specific cases. Cats living on the island, for example, must have a guardian, be neutered, individually identified, and cannot roam the island without restraint (for example, the cat must be on a leash) [169,170].

## 6. Thinking About Animal Welfare: Future Directions in Brazil

When considering lethal methods for controlling free-roaming cats, serious ethical concerns emerge regarding the following dilemma: healthy animals are killed to mitigate their negative impacts on biodiversity. How can we effectively manage these cats while simultaneously ensuring their welfare? From an ethical standpoint, considering each animal as a sentient being deserving of life, such practices may be deemed unacceptable. Animal welfare encompasses the mental and physiological state of an individual in relation to its environment [171]. Good welfare requires disease prevention, appropriate veterinary care, shelter, management, nutrition, a stimulating and safe environment, humane handling, and humane killing [172,173,174].

According to the Terrestrial Animal Health Code, “Animals with serious health problems should be isolated and treated promptly or humanely euthanized if treatment is not feasible or recovery is unlikely” [175]. In Brazil, Article 225 of the Federal Constitution prohibits any practice that subjects animals to cruelty [176]. Additionally, Article 32 of the Environmental Crimes Law (Law 9605) criminalizes acts of abuse, mistreatment, harm, or mutilation of wild, domestic, or domesticated animals, whether native or exotic [177]. In 2020, this law was amended to increase penalties, establishing imprisonment ranging from 2 to 5 years and the prohibition of animal custody when the mistreated animals are dogs and cats. Furthermore, Law 14,228, sanctioned in 2021, prohibits euthanasia of stray dogs and cats, except in cases of serious illness or incurable infectious diseases [166].

The Environmental Crimes Law [177] and Resolution 1044/2013 from the Federal Council of Veterinary Medicine [178] regulate euthanasia procedures in Brazil. These regulations stipulate that euthanasia should only be performed by veterinarians, and only when an animal is suffering profoundly due to severe injury, chronic or severe disease, advanced age, or serious behavioral problems. Furthermore, euthanasia must be conducted using methods and techniques that minimize pain. Regarding lethal methods of cat control, most are not fully effective and cause unnecessary suffering, even in animals without prior illness [179]. For instance, if an animal does not consume enough bait to cause immediate death or may inadequately absorb the toxic substance through licking. Similarly, shooting may fail to hit vital anatomical areas, prolonging distress.

The majority of studies on the efficacy of control methods focus on reporting that the method used is specific to cats, such as encapsulated para-aminopropiophenone (PAPP) baits. This substance is highly toxic to cats and dogs. Encapsulated baits pass directly into a cat’s stomach, due to its behavior of not chewing, unlike other native mammals that tend to chew and discard hard food items. Cats are easily attracted to and metabolize the poison. However, there is little information reporting how death occurs, including the interval from ingestion of the bait to death and observed behaviors. Some authors argue that poisoning could be considered “humane” [180]. However, in another study the humaneness of poisoning was rated as “mild suffering” by using a relative humaneness model, with a delay of 185 min from the first definitive sign to death [181].

Mild suffering is defined by Sharp–Saunders [181] as when “Loss of consciousness is not immediate and there is no or only minimal aversion and no or only mild suffering before death;” this means exhibiting clinical signs such as lethargy, emesis, and dyspnea [181]. In a study by Johnston et al. [182], all 30 poisoned cats had lethargy and reduced respiration, and 7 of the 30 exhibited emesis before death [182]. Other clinical signs related to poisoning were observed in a smaller number of cats, including excessive salivation, vocalizations, and seizures [182]. Beyond physical issues, despite some behaviors being considered “mild”, they still indicate negative mental states, such as distress and/or pain and fear [181], which impede the animal from engaging in its normal behavioral repertoire [183].

The cat-shooting method is defined as “Direct destruction or concussion of brain tissue resulting in rapid unconsciousness” [181]. It is considered a method that causes no suffering but only when performed with accuracy (e.g., a precise shot to the head) [181]. However, another study reported serious welfare issues related to this practice, including the emergence of mutilated and injured cats due to “culling by firearms” programs conducted in the Port of Newcastle, Australia [184]. Many people protested against continuation of this practice, expressing concerns about animal welfare [184].

Cat caregivers also experience negative mental states when faced with lethal control strategies. When researchers interviewed a female caregiver about the cats in the Port of Newcastle, she remarked: “…they’re basically the same as a pet cat that you’d have at home. They have names. They have personalities. They have their little traits that they each individually have…” People are concerned about animal welfare, but human welfare is less frequently addressed. Studies report a higher incidence of negative mental states, including stress, depression, and even suicide, among individuals who must euthanize cats in shelters [185,186]. The welfare of these individuals is also affected by exposure to lethal control programs for cats. In a study where caregivers were interviewed about their feelings regarding cat culling in Newcastle, immediate emotional impacts such as trauma, disbelief, shock, and grief, as well as long-term psychological impacts, e.g., complicated grief, betrayal, pervasive distrust, and post-traumatic stress disorder symptoms, were observed [187].

For many people, cats are considered members of the family. Some guardians form strong bonds with their pets, perceiving them as unique beings and developing a sense of empathy towards them [188]. Empathy is defined as the ability to emotionally connect with animals and to act with compassion and respect towards them [189,190]. This capacity often begins early in life; for instance, children who had pets during their childhood tend to exhibit greater empathy and engage in more positive behaviors towards dogs and cats [191].

Human–cat interactions can enhance welfare for both parties, leading to reductions in cardiovascular disease [192], negative moods [193]. Additionally, the presence of a cat can decrease feelings of loneliness [194] in their guardian and also induce positive mental states, such as feeling more relaxed as a result of the cat’s purring [193]. Conversely, cats benefit from increased veterinary care, proper nutrition, and engaging in positive interactions with their environment and caregivers [34]. Lethal methods used for controlling cats, including those without guardians, are often viewed unfavorably by individuals who empathize with these animals. As such, these practices require further investigation focused on feline welfare before they can be broadly accepted.

Various studies have assessed the welfare implications of wildlife control methods, indicating that they should be chosen based on the species, environment, and context to avoid lethal animal suffering [194,195,196,197,198]. Nevertheless, all vertebrates can experience pain and distress, regardless of whether they are considered pests. This represents a significant omission in the literature on the effects of cat control measures on welfare, and further scientific effort is needed to better understand this issue from a global perspective. Furthermore, studies in conservation and animal welfare are not entirely opposed; there are points of convergence. For example, both wildlife conservationists and welfare advocates should be interested in cat-friendly control measures. Therefore, there are opportunities for constructive engagement between wildlife conservation and animal welfare organizations [199].

In Brazil, there is limited knowledge about cat-control strategies and even less understanding of how these strategies could affect cats’ welfare. A widely used strategy to control populations of stray dogs and cats is TNR, mainly from a public health perspective. Recently, an Action Plan for Cat Control was implemented in Fernando de Noronha (see Section 5.1.2), with the following objectives: reduce feral cats’ population, control semi-domiciled cats’ population, raise awareness in society about impacts of cats on biodiversity and public health, and continuously monitor cats and other introduced animals. To this end, cats were captured, neutered, and returned with a chip to enable monitoring.

In summary, for future directions, this literature review concludes that there are insufficient scientific data regarding impacts of cats on wildlife in Brazil. Therefore, we assert the need for more research into cat management strategies. For example, funding is needed to acquire basic monitoring equipment. With acquisition and installation of such equipment, cats could be monitored in vulnerable areas for native wildlife, e.g., reserves for biodiversity. More comprehensive studies are essential to generate robust databases and draft realistic and effective management strategies, and to ensure that public institutions understand the scale of the problem and establish effective partnerships. Conservation units can include the issue of invasive alien species in their management plans and seek solutions together with ministries and research institutions, as in the case of Fernando de Noronha. In Brazil, even in very compromised areas, such as island environments, the strategy of TNR has been used, but its effectiveness remains uncertain, and more long-term studies are needed to assess costs and benefits. As there are relatively few data in the literature from Brazil, it is not possible yet to accurately estimate impacts of cats on wildlife or on disease transmission.

Managing free-roaming cat populations comprehensively through sterilization, microchipping, and responsible adoption emerges as a multifaceted potential solution to ecological and animal welfare challenges. These measures not only control the number of cats but also promote their well-being by increasing opportunities for responsible adoption [200]. Additionally, the recommendation to keep cats indoors [137] further reinforces the reduction in ecological impacts and improves animal welfare. This practice advocates responsible pet ownership and the protection of native wildlife.

Finally, when managing stray and/or feral cats, it is important to consider both community concerns and the welfare of individual cats. When discussing cats’ responsible ownership, it is important to include all stakeholders, such as ecologists, veterinarians, and politicians, in addition to cat guardians. Topics to include in the aforementioned discussion with relevant stakeholders may include, but not be limited to, veterinary services such as sterilization, rehabilitation, and behavioral advice, creation or enhancement of a rehoming and fostering network, and the need for community involvement. These strategies should include a feral cat program supported by volunteers, public education, and information dissemination through tools such as marketing. All activities and methods used must consider “best practices” in animal welfare.

## 7. Conclusions

As a species, cats are neither “good” nor “bad;”rather, they are a domesticated species that remains opportunistic carnivores, even when they are fed regularly. Cats can be both an avid hunter and a beloved pet. Whether a beloved pet, a cat that has strayed from home, or one that was born feral, it can not only prey upon wildlife but also transmit disease(s) to both wildlife and humans. Some researchers believe that neutering strategies should be encouraged, but in countries where cat impacts are severe, lethal strategies have been used to control cat populations, raising ethical concerns about cat welfare. In Brazil, the situation is quite complex as, in addition to the lack of studies on cat impacts, basic monitoring equipment such as camera traps and radio collars is rarely available in natural areas such as conservation units and parks. This scenario is a consequence of several factors, including the scarcity of resources and limited financial support for research in Brazil and other developing-world nations. Impacts of cat predation on wildlife may be greater than previously documented areas of the globe where studies are scarce. Because the problem is related to free-roaming cats, it is important that people understand the risks and benefits associated with any selected management strategy. The key to the challenge for all stakeholders may lie in cooperation, awareness, and, above all, how these control managements may affect the welfare of cats, wildlife, and people.

## Supplementary Materials

The following supporting information can be downloaded at: https://www.mdpi.com/article/10.3390/ani15020190/s1, Table S1: Selected articles with “cat” as a key species related to their impacts on wildlife (e.g., predation, spatial overlap, or disease transmission) in Brazil.
