# Supplementary material for: The Wildcat That Lives in Me: A Review on Free-Roaming Cats (Felis catus) in Brazil, Focusing on Research Priorities, Management, and Their Impacts on Cat Welfare
Source: Animals (Basel). 2025 Jan 12;15(2):190. doi: 10.3390/ani15020190 (PMC11759153; doi:10.3390/ani15020190)
Supplement: Supplementary file 1 [file animals-15-00190-s001.zip › animals-3358897-supplementary.pdf]

Supplementary material

Table S1. Selected articles with "cat" as a key specie related to their impacts on wildlife (e.g. predation, spatial overlap or disease transmission) in Brazil.

	Author and year	*City/State of Brazil*	*Environment*	*Biome*	*Cat-Prey type*	*Methodology*	*Cat-Impact*
**1.**	**André et al. 2014**	São Paulo, São Paulo	Continent	Atlantic Forest	-	Molecular test (PCR)	Disease transmission: Anaplasma spp; Mycoplasma haemofelis; Candidatus Mycoplasma; haemominutum; Candidatus Mycoplasma turicensis; Bartonella spp.; Babesia vogeli and Theileria spp.
**2.**	**Benavides et al. 2020**	Brazil	Continent (rural and urban)	All	-	Data and Spatio-temporal analyses	Disease transmission: Rabies
**3.**	**Braga et al. 2012**	São Luís, Maranhão	Continent	Atlantic Forest	-	Serological Technique (Indirect Fluorescence AntibodyTest - IFAT)	Disease transmission: Toxoplama Gondii and Neospora Caninum
**4.**	**Bresciani et al. 2007**	Araçatuba, São Paulo	Continent	Atlantic Forest	-	Serological Technique (Indirect Fluorescence AntibodyTest - IFAT)	Disease transmission: Toxoplama Gondii and Neospora Caninum
**5.**	**Campos et al. 2007**	Piracicaba, São Paulo	Continent	Atlantic Forest	Mammals	Scat collection and Analysis	Predation
**6.**	**Coelho et al. 2011**	Andradina, São Paulo	Continent	Atlantic Forest	-	Serological Technique (Indirect Fluorescence AntibodyTest - IFAT)	Disease transmission: Toxoplama Gondii, Neospora Caninum and Leishmania spp.
**7.**	**Costa et al. 2012**	Fernando de Noronha, Pernambuco	Island	Atlantic Forest	-	Serological Technique	Disease transmission: Toxoplasma Gondii
**8.**	**Costa-Pinto et al. 2020**	Maceió, Alagoas	Continent/urban	Atlantic Forest	Bat (Phyllostomus discolor)	Night Watchmen record	Predation
**9.**	**Da Silva et al. 2008**	Rio de Janeiro, Rio de Janeiro	Continent	Atlantic Forest		Molecular test (PCR)	Disease transmission: American visceral leishmaniosis (25).
**10.**	**Dias et al. 2017**	Fernando de Noronha, Pernambuco	Island	Atlantic Forest		Distance sampling and Questionnaire survey	Cat density

11.	**Ferreira et al. 2014**	Ilha Comprida, São Paulo	Island	Atlantic Forest	Mammals Vertebrates: (76.6); Birds (22.1) birds and amphibians (1.3). Invertebrates: Insects (93.6); crustaceans (5.3) and mollusks (1.1).	Fecal sample collection, Footprint and Scarification analysis	Predation	
12.	**Ferreira et al. 2017**	Ilha Comprimida, São Paulo	Island	Atlantic Forest	Invertebrates (58), Mammals (33), Birds (10), Amphibians (0.4).	Fecal sample collection and Analysis	Predation	
13.	**Ferreira et al. 2018**	Ilha Comprida, São Paulo	Island	Atlantic Forest	-	Radio-telemetry, Location of feces, Traces and Camera Traps	Spatial Overlap	-
14.	**Ferreira et al. 2019**	Ilha Comprida, São Paulo	Island	Atlantic Forest	Mammals		Predation	
15.	**Fontalvo et al. 2017**	Petrolina and Lagoa Grande, Pernambuco	Continent	Caatinga	-	Molecular (PCR) and Serological technique (Indirect Fluorescence AntibodyTest - IFAT)	Disease transmission: Barrtonella	
16.	**Fournier et al. 2014**	Natal, Rio Grande do Norte	Continent	Atlantic Forest	-	Serological (Modified Agglutination Test - MAT) Molecular and the Mouse-bioassay techniques	Disease Transmission: Toxoplama Gondii Serological (52.8); Molecular (8.1); mouse-bioassay techniques (0).	
17.	**Furtado et al. 2013**	Goiás, Mato Grosso do Sul and Tocantins.	Continent	Brazilian Cerrado, Pantanal and Amazon	-	Serological Technique and FeLV Screening test	Disease transmission prevalence: Canine distemper virus (0); Rabies virus (20) at Pantanal and (11) at Cantão State Park; Feline immunodeficiency virus antibodies (0), and Feline leukemia virus antigen (15).	
18.	**Furtado et al. 2015**	Goiás, Mato Grosso do Sul and Tocantins.	Continent	Brazilian Cerrado, Pantanal and Amazon	-	Serological Technique (Modified Agglutination Test – MAT)	Disease transmission prevalence (%) Leptospira spp. and Toxoplasma gondii	
19.	**Lessa et al. 2012**	Ilha Grande, Rio de Janeiro	Island	Atlantic Forest	Invertebrates (54), mammals (18), birds (11), reptiles	Prey collected by cat owners	Predation	

				(6) and amphibians (4).		
**20. Lima et al. 2017**	Fernando de Noronha, Pernambuco	Island	Atlantic Forest	-	Fecal sample collection and analysis	Diasese Transmission: Ancylostoma sp., Strongyloides sp., Trichuris campanula and Toxocara cati
**21. Lima et al. 2018**	Fernando de Noronha, Pernambuco	Island	Atlantic Forest	-	Serological technique (Neospora agglutination test - NAT)	Disease Transmission: Neospora Caninum (3.11)
**22. Magalhães et al. 2017**	Fernando de Noronha, Pernambuco	Island	Atlantic Forest	-	Serological technique (Indirect Fluorescence AntibodyTest - IFAT)	Diasese Transmission: Toxoplasma Gondii<br>Pet cats (71.26)<br>Feral Cats (54.74)
**23. Massara et al. 2016**	Minas Gerais	Continent	Atlantic Forest	-	Camera traps	Spatial Overlap and resource competition
**24. Melo et al. 2016**	Fernando de Noronha, Pernambuco	Island	Atlantic Forest	-	Molecular (PCR), Serological Technique (Indirect Fluorescence AntibodyTest - IFAT) and Mouse Bioassay	Disease Transmission: Toxoplasma Gondii
**25. Sevá et al. 2018**	Teodoro Sampaio, São Paulo	Continent/rural	Atlantic Forest	-	Fecal sample collection and Analysis	Disease Transmission
**26. Cavalcante et al. 2006**	Rôndonia	Continent	Amazon	-	Serological Technique (IFAT and MAT).	Disease Transmission: Toxoplasma Gondii
**27. Dalla Rosa et al. 2010**	Santa Catarina	Continent	Atlantic Forest	-	Serological Technique (Indirect Fluorescence AntibodyTest - IFAT)	Disease Transmission: Toxoplasma Gondii
**28. Dubey et al. 2002**	Guarulhos, São Paulo	Continent	Atlantic Forest	-	Direct Agglutination tests	Disease Transmission: Neospora caninum and Sarcocystis neurona
**29. Pena et al. 2006**	São Paulo	Continent	Atlantic Forest	-	Serological, Molecular, Mouse assay and Oocyst shedding	Disease Transmission: Toxoplasma Gondii
**30. Dubey et al. 2004**	Paraná	Continent	Atlantic Forest	-	Serological, Molecular, and Mouse assay	Disease Transmission: Toxoplasma Gondii
**31. Silva et al. 2002**	Guarulhos, São Paulo	Continent	Atlantic Forest	-	Serological Technique (MAT)	Disease Transmission: Toxoplasma Gondii
**32. Onuma et al., 2014**	Mato Grosso	Continent	Pantanal	-	Serological Technique (Indirect Fluorescence AntibodyTest - IFAT)	Disease Transmission: Toxoplasma Gondii, N. caninum e S. neurona
**33. Silva et al., 2001**	78 cities in 20 states - Brazil	Continent	All	-	Modified Agglutination test	Disease Transmission: Toxoplasma Gondii
**34. Filoni et al., 2006**	Rondônia, Acre, Mato Grosso, Mato Grosso do Sul,	Continent	Amazon Forest, Atlantic Forest,		Serological Technique (Indirect Fluorescence	Disease Transmission: Toxoplasma Gondii; Feline herpesvirus 1, calicivirus, coronavirus, parvovirus, Ehrlichia

	Paraná, São Paulo e Rio de Janeiro – Brazil.	Cerrado, and Pantanal.	AntibodyTest - IFAT), Western blot and ELISA.	canis, Anaplasma phagocytophilum,and Bartonella henselae.
